# Regulation of Antibiotic Production by Signaling Molecules in *Streptomyces*

**DOI:** 10.3389/fmicb.2019.02927

**Published:** 2019-12-19

**Authors:** Dekun Kong, Xia Wang, Ju Nie, Guoqing Niu

**Affiliations:** ^1^Biotechnology Research Center, Southwest University, Chongqing, China; ^2^State Cultivation Base of Crop Stress Biology for Southern Mountainous Land, Academy of Agricultural Sciences, Southwest University, Chongqing, China; ^3^College of Horticulture and Landscape Architecture, Southwest University, Chongqing, China

**Keywords:** antibiotic biosynthesis, regulation, *Streptomyces*, hormone-like signaling molecule, antibiotic biosynthetic intermediate, elicitor

## Abstract

The genus *Streptomyces* is a unique subgroup of actinomycetes bacteria that are well-known as prolific producers of antibiotics and many other bioactive secondary metabolites. Various environmental and physiological signals affect the onset and level of production of each antibiotic. Here we highlight recent findings on the regulation of antibiotic biosynthesis in *Streptomyces* by signaling molecules, with special focus on autoregulators such as hormone-like signaling molecules and antibiotics themselves. Hormone-like signaling molecules are a group of small diffusible signaling molecules that interact with specific receptor proteins to initiate complex regulatory cascades of antibiotic biosynthesis. Antibiotics and their biosynthetic intermediates can also serve as autoregulators to fine-tune their own biosynthesis or cross-regulators of disparate biosynthetic pathways. Advances in understanding of signaling molecules-mediated regulation of antibiotic production in *Streptomyces* may aid the discovery of new signaling molecules and their use in eliciting silent antibiotic biosynthetic pathways in a wide range of actinomycetes.

## Introduction

The genus *Streptomyces*, a unique subgroup of actinomycetes bacteria, is best-known for their ability to produce an enormous variety of bioactive secondary metabolites including antibiotics. The onset and production level of each antibiotic is subject to complex control by various environmental and physiological signals (Liu et al., 2013; Niu et al., 2016). Several excellent and comprehensive reviews have focused on the roles of signaling molecules in the regulation of antibiotic production of *Streptomyces* species (Takano, 2006; Sidda and Corre, 2012; Niu et al., 2016; Okada and Seyedsayamdost, 2017). Hormone-like signaling molecules are a group of small diffusible signaling molecules that can elicit antibiotic production and/or induce morphological differentiation at nanomolar concentrations. Typically, a hormone-like signaling molecule binds to its specific receptor, and exerts regulatory function through regulators at different hierarchical levels, including global regulators, pleiotropic regulators, and cluster-situated regulators (CSRs) (Liu et al., 2013; Niu and Tan, 2015; Wei et al., 2018). The most-studied regulatory system is the A-factor cascade that involves the signaling molecule A-factor and its receptor ArpA necessary for streptomycin and grixazone production in *Streptomyces griseus* (Horinouchi, 2007; Horinouchi and Beppu, 2007). Aside from the hormone-like signaling molecules, accumulating evidence suggests that antibiotics and their biosynthetic intermediates can also serve as autoregulators to modulate their own biosynthesis and as cross-regulators of disparate biosynthetic pathways (Niu et al., 2016). This review is not intended to be comprehensive, but to highlight recent findings on the regulation of antibiotic biosynthesis in *Streptomyces* by small molecules including hormone-like signaling molecules and antibiotics/their biosynthetic intermediates. A better understanding of the regulatory cascade of antibiotic production by signaling molecules in *Streptomyces* may aid the discovery of new signaling molecules and speed up their application in eliciting silent antibiotic biosynthetic pathways in actinomycetes.

## Representative Hormone-Like Signaling Molecules Controlling Antibiotic Biosynthesis

Hormone-like signaling molecules are diffusible small molecules that can elicit antibiotic production and/or induce morphological differentiation at nanomolar concentrations. They exert their regulatory effect via specific receptor proteins that usually belong to the TetR family of transcriptional regulators (Takano, 2006; Niu et al., 2016). The TetR family regulators are characterized by a N-terminal helix-turn-helix (HTH) DNA-binding motif and a C-terminal ligand regulatory domain. They are widely distributed among bacteria and regulate diverse cellular processes in bacteria, especially antibiotic biosynthesis in *Streptomyces* (Cuthbertson and Nodwell, 2013). In *Streptomyces* species, A-factor, the first signaling molecule to be discovered, induces streptomycin production and morphological differentiation through a regulatory cascade involving the receptor ArpA, the pleiotropic regulator AdpA, and the CSR activator StrR in *S. griseus* (Horinouchi, 2007; Horinouchi and Beppu, 2007). Inspired by studies on the A-factor regulatory cascade, many more hormone-like signaling molecules have been identified, and great efforts has been directed to understanding signaling molecules-mediated regulatory cascades of antibiotic biosynthesis in *Streptomyces*.

### Five Major Classes of Hormone-Like Signaling Molecules

**FIGURE 1:**
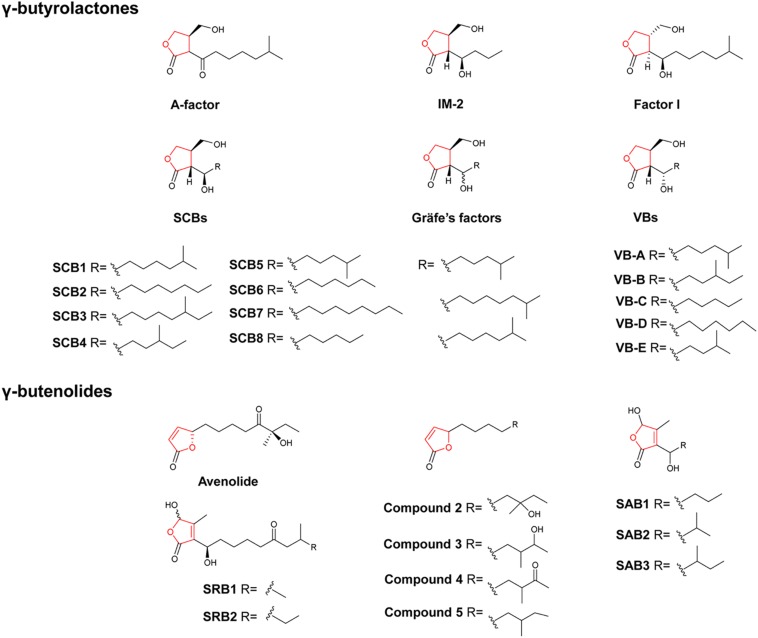
Chemical structures of representative γ-butyrolactones and γ-butenolides. Compounds 2–5: the four γ-butenolides from *Streptomyces albus* J1074.

Similar to the well-documented *N*-acyl-homoserine lactones (AHLs) in Gram-negative bacteria, hormone-like signaling molecules in the Gram-positive *Streptomyces* bacteria are diffusible low molecular weight chemical substances that can elicit antibiotic production and/or induce morphological differentiation at nanomolar concentrations (Swift et al., 1994; Takano, 2006). To date, hormone-like signaling molecules identified from *Streptomyces* are classified into five major classes, including γ-butyrolactones (GBLs), furans, γ-butenolides as well as PI factor and *N*-methylphenylalanyl-dehydrobutyrine diketopiperazine (MDD) (Niu et al., 2016). GBLs, furans and γ-butenolides are based on five-membered heterocyclic rings containing four carbons and one oxygen, while PI factor and MDD have quite different structures (Recio et al., 2004; Matselyukh et al., 2015). As most recent investigations are largely on GBLs and γ-butenolides, we herein only summarize recent findings on these two important classes. GBLs, the largest group of these signaling molecules, share a characteristic 2,3-disubstituted GBL core skeleton but differ in the length, branching and stereochemistry of the acyl side chain (Takano, 2006). So far, a total of 19 GBLs have been identified in streptomycetes, including the A-factor from *S. griseus*, eight butanolides (SCB1-8) from *Streptomyces coelicolor*, five virginiae butanolides (VBs A–E) from *Streptomyces virginiae*, IM-2 from *Streptomyces lavendulae*, factor 1 from *Streptomyces viridochromogenes*, and three Gräfe’s factors from *Streptomyces bikinensis* and *Streptomyces cyaneofuscatus* (Figure 1; Niu et al., 2016; Sidda et al., 2016). To gain insight to the distribution of GBLs, an A-factor-deficient mutant of *S. griseus* was used as an indicator strain to screen against 203 actinomycete strains. Thirty of the strains tested showed distinct A-factor activity, indicating that A-factor was widely distributed among actinomycetes (Hara and Beppu, 1982). Earlier studies identified the butenolide synthase AfsA as a key enzyme for A-factor biosynthesis in *S. griseus*, and the AfsA homolog ScbA was required for the biosynthesis of the three SCBs in *S. coelicolor* (Takano et al., 2001; Hsiao et al., 2007). Among the eight butanolides identified in the model organism *S. coelicolor*, SCB1-3 were identified in the 2000s (Takano et al., 2000; Hsiao et al., 2009). Recently, the engineered strain *S. coelicolor* M1152 was found to overproduce GBLs SCB1–3 as well as five novel GBLs designated as SCB4–8 (Sidda et al., 2016). The *S. coelicolor* M1152 is a derivative of *S. coelicolor* M145 lacking four biosynthetic gene clusters (BGCs) for the biosynthesis of actinorhodin (ACT), undecylprodigiosin (RED), calcium-dependent antibiotic (CDA) and coelimycin. It should be noted that the deletion of the coelimycin gene cluster included *scbR2*, which encodes a repressor of SCBs biosynthetic gene *scbA*, and thereby contributed to the overproduction of SCBs in *S. coelicolor* M1152 (Gomez-Escribano and Bibb, 2012; Sidda et al., 2016). In a BLAST survey, we found one to three *afsA*-like genes in each of nine *Streptomyces* genomes (Niu et al., 2016). In another study, BLAST search for homologs of AfsA from *S. griseus* within the actinobacteria genomes available from public databases showed that AfsA-like proteins are present in most actinomycetes (Ahmed et al., 2017). The widespread distribution of GBLs was further confirmed by using VB- and IM-2-deficient strains as indicators to examine VB and IM-2 distribution. A total of 10 strains of the 40 *Streptomyces* and 11 endophytic actinomycetes tested produce IM-2, and the same number of strains produced VB active compounds (Thao et al., 2017). These studies reinforce the notion that GBLs are the most common hormone-like signaling molecules among actinomycetes. More efforts should be directed to identifying chemical structures of these signaling molecules.

Representative γ-butenolides include two butenolides (SRB1 and SRB2) from *Streptomyces rochei* (Arakawa et al., 2012; Arakawa, 2014), the avenolide from *Streptomyces avermitilis* (Kitani et al., 2011), four butenolides from *Streptomyces albus* J1074 (Nguyen et al., 2018), and three butenolides (SAB1-3) from *Streptomyces ansochromogenes* (Wang et al., 2018; Figure 1). Of special note is the avenolide that is necessary for triggering avermectin production in *S. avermitilis* (Kitani et al., 2011). The gene cluster responsible for avenolide biosynthesis consists of three genes (*avaR1-3*) that encode GBL receptor homologs, and two genes (*aco* and *cyp17*) that encode an acyl-CoA oxidase and a cytochrome P450 hydroxylase, respectively. Studies suggested that both the acyl-CoA oxidase Aco and the cytochrome P450 hydroxylase Cyp17 were required for the enzymatic production of avenolide. Furthermore, Aco/Cyp17 homologs were also found in the genomes of *Streptomyces fradiae*, *Streptomyces ghanaensis*, *Streptomyces griseoauranticus*, and *S. albus* J1074, suggesting that they have the capacity to produce avenolide or avenolide-like autoregulators (Kitani et al., 2011; Ahmed et al., 2017). Recently, Luzhetskyy and colleagues identified an avenolide-like compound 4 in a genetically modified strain of *S. albus* 1074 (Ahmed et al., 2017). In another study, Nihira and colleagues investigated the distribution of avenolide by using a *S. avermitilis aco* disruptant as a biosensor in a collection of 40 *Streptomyces* and 11 endophytic actinomycetes. They showed that 12 of the 51 actinomycetes strains exhibited avenolide activity with *S. albus* J1074 showing the highest activity (Thao et al., 2017). Metabolite profiling of a disruptant of the *S. albus aco* gene led to the identification of the compound 4 along with three other butenolides (compounds 2, 3, and 5). The four avenolide-like compounds showed different levels of avenolide activity in stimulating avermectin production in *S. avermitilis* (Nguyen et al., 2018). It is interesting to note that the four compounds have been isolated previously from marine-derived *Streptomyces* strains, though there are no reports on their connection with antibiotic biosynthesis in the native producers (Cho et al., 2001; Strand et al., 2014; Viegelmann et al., 2014; Igarashi et al., 2015). In a recent study, Tan and colleagues identified a putative BGC for autoregulator biosynthesis in *S. ansochromogenes* (nikkomycin producer). Within the gene cluster, *sabA* encodes an AfsA-like enzyme, whereas *sabP* and *sabD* encode phosphatase and dehydrogenase enzymes, respectively. Heterologous expression of *sabAPD* in *E. coli* and *Streptomyces* led to the identification of three novel butenolides (SAB1, 2, and 3) (Wang et al., 2018). Since there are only limited numbers of natural GBLs and γ-butenolides identified in *Streptomyces*, these studies provide effective strategies for the discovery of new signaling molecules, which are normally produced in very small quantities. It is noteworthy that several butenolides have also been identified in *Streptomyces* species (Wang T. et al., 2014; de Oliveira et al., 2019), especially from marine-derived *Streptomyces* (Cho et al., 2001; Strand et al., 2014; Viegelmann et al., 2014; Igarashi et al., 2015). The roles of these butenolides in their native producers await further investigation.

### Receptors of Hormone-Like Signaling Molecules

**FIGURE 2:**
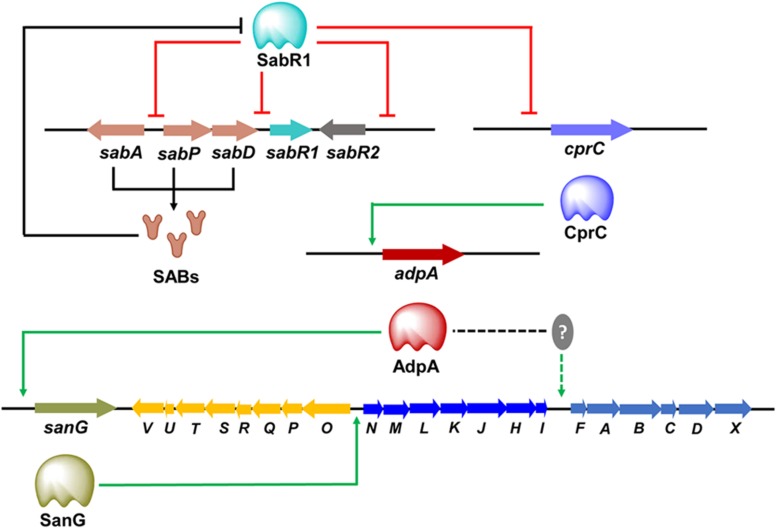
Cascade regulation of nikkomycin biosynthesis. The SABs synthesized by SabA, SabP, and SabD exert regulatory functions via the receptor SabR1. SabR1 represses the transcription of *cprC* and other target genes (*sabA*, *sabR1*, *sabR2*) by binding directly to their promoter regions. Binding of SABs to SabR1 causes the dissociation of SabR1 from *cprC* promoter, and thereby releases its repression on the transcription of *cprC*, which in turn activates *adpA* transcription to stimulate nikkomycin production. AdpA activates nikkomycin biosynthesis via activating the transcriptional initiation of *sanG*. SanG promotes nikkomycin biosynthesis through directly binding to the bidirectional *sanN*–*sanO* promoter region and activating the transcription of the biosynthetic genes. AdpA can also activate the transcription of *sanF-X* through an unknown mediator.

**FIGURE 3:**
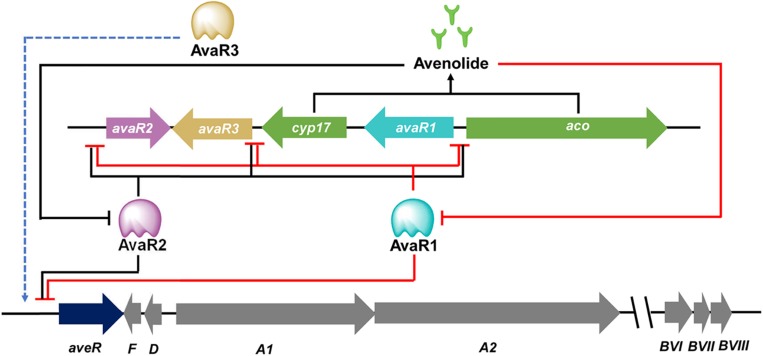
Cascade regulation of avermectin biosynthesis. The complex regulation of avermectin production involves three GBL receptors. The avenolide receptor AvaR1 inhibits avenolide and avermectin production by directly repressing the transcription of *aco*, *aveR*, *avaR1*, *avaR2*, and *avaR3*. The pseudo GBL receptor AveR2 also acts as a repressor of avenolide and avermectin biosynthesis by binding to the same targets as AveR1. Another GBL receptor AvaR3 promotes avermectin production through an unknown regulatory mechanism. The DNA binding activities of AvaR1 and AvaR2 are modulated by avenolide, which are the enzymatic product of Aco and Cyp17.

The transmission of chemical signals starts with the binding of an autoregulator to its specific receptor protein, that usually belongs to the TetR family of transcriptional regulators. It is not uncommon that many *Streptomyces* genomes contain multiple genes for ArpA-like GBL receptors. Examples include ScbR, ScbR2, CprA and CprB in *S. coelicolor*, JadR2 and JadR3 in *Streptomyces venezuelae*, and AvaR1, AvaR2, and AvaR3 in *S. avermitilis*, SabR1 and SabR2 in *S. ansochromogenes*. It is noteworthy that most genes encoding these GBL receptors are closely linked both to each other and to the *afsA*-like genes, except that *cprA* and *cprB* are not linked to an *afsA*-like gene and therefore referred to as “orphan GBL receptor” genes (Onaka et al., 1998). To date, only a few identified receptors have been shown to interact with endogenous GBL molecules. In the model organism *S. coelicolor*, ScbR was characterized as a genuine receptor of SCBs (Takano et al., 2005; Gomez-Escribano et al., 2012). ScbR binds the *scbR*-*scbA* intergenic region to repress its own expression, while interacting with ScbA to activate *scbA* expression in response to SCBs (Takano et al., 2001). Another known target of ScbR is *kasO*, the CSR activator of the coelimycin biosynthetic pathway (Takano et al., 2005). In *S. venezuelae*, JadR3 was identified as a genuine receptor of SVB1, a GBL identical in structure to SCB3 of *S. coelicolor*. The SVB1 receptor JadR3 has dual activation and repression effects on jadomycin biosynthesis (Zou et al., 2014; Niu et al., 2016). In *S. ansochromogenes*, SabR1, the cognate receptor of SABs, was shown to repress the expression of *sabA*, *sabR1*, *sabR2* and *cprC* by binding directly to their promoter regions. Interestingly, CprC, a homolog of CprA and CprB in *S. coelicolor*, was identified as an activator of the pleiotropic regulatory gene *adpA*, which in turn stimulated nikkomycin biosynthesis via activating the transcriptional initiation of the CSR activator gene *sanG* and biosynthetic genes of the *sanF*-*X* operon (Figure 2; Pan et al., 2009; Wang et al., 2018). Of the three GBL receptors in *S. avermitilis*, AvaR1 acts as an avenolide receptor to control avenolide and avermectin production by directly repressing the transcription of *aco*, *aveR*, its own gene and the other two GBL receptor homologous genes (*avaR2* and *avaR3*) (Figure 3; Zhu et al., 2017). ScbR2, JadR2, AvaR2 and SabR2 were identified as pseudo GBL receptors (Xu et al., 2010; Zhu et al., 2016; Wang et al., 2018), and the roles of these pseudo GBL receptors in the regulation of antibiotic biosynthesis will be discussed in a subsequent section. AvaR3 contains an extra 75-amino acid stretch that is not present in typical GBL receptors, and promotes avermectin production through a yet-unknown regulatory mechanism (Miyamoto et al., 2011). It is interesting to note that AvaR3 may represent a new subgroup of GBL receptors, and therefore further studies are needed to reveal its role in avermectin production.

## Antibiotics as Regulatory Ligands Regulating Antibiotic Biosynthesis

Other than the hormone-like signaling molecules described above, antibiotics and their biosynthetic intermediates can also serve as regulatory ligands to regulate their own biosynthesis or cross-regulators of disparate biosynthetic pathways. In jadomycin biosynthesis, jadomycin and its biosynthetic intermediates actively participate in both feedback and feedforward cascade control of their own production in *S. venezuelae.* Such interplay makes jadomycin biosynthesis an illuminating model for regulatory studies of antibiotic production mediated by antibiotics and their biosynthetic intermediates (Niu et al., 2016). Aside from roles in regulating their own biosynthesis, antibiotics can also serve as autoregulators to control disparate antibiotic biosynthetic pathways. Typically, antibiotics exert their regulatory effect through CSRs that are associated with BGCs or pleiotropic regulators situated elsewhere in the genome (Table 1).

**TABLE 1:** Representative regulators of antibiotic biosynthesis with antibiotics and/or their biosynthetic intermediates as regulatory ligands.

**Regulators**	**Ligands**	**Description**	**References**
JadR1	JdB, JdA, DHU, DHR	TetR family, activator of jadomycin biosynthesis; repressor of chloramphenicol biosynthesis	Wang et al., 2009
RedZ	RED	TetR family, activator of RED biosynthesis	Wang et al., 2009
JadR^∗^	DHU, DHR, JdA, JdB	TetR family, repressor of jadomycin biosynthesis	Zhang et al., 2013
ChlF1	CHL, DM-CHL, deschloro-CHL	TetR family, repressor of chlorothricin biosynthesis	Li et al., 2016
CalR3	calcimycin, cezomycin	TetR family, repressor of calcimycin biosynthesis	Gou et al., 2017
NosP	NOS, NOS-AC	SARP family, activator of nosiheptide production	Li et al., 2018
RifQ	rifamycin B	TetR family, repressor of rifamycin biosynthesis	Lei et al., 2018
PhlH	2,4-DAPG, MAPG	TetR family, repressor of 2,4-diacetylphloroglucinol biosynthesis	Yan et al., 2017
ScbR2	ACT, RED	TetR family, repressor of coelimycin biosynthesis	Xu et al., 2010
JadR2	JdB, Cm	TetR family, repressor of jadomycin biosynthesis; activator of chloramphenicol	Xu et al., 2010

*JdB, jadomycin B; JdA, jadomycin A; DHU, 2,3-dehydro-UWM6; DHR, dehydrorabelomycin; RED, undecylprodigiosin; CHL, chlorothricin; DM-CHL, demethylsalicycloyl chlorothricin; deschloro-CHL, deschloro-chlorothricin; NOS, nosiheptide; NOS-AC, the intermediate of NOS; 2,4-DAPG, 2,4-diacetylphloroglucinol; MAPG, monoacetylphloroglucinol; ACT, actinorhodin; Cm, chloramphenicol.*

### Antibiotics and Their Biosynthetic Intermediates as Autoregulators of Their Own Biosynthesis

The first line of antibiotics as autoregulators came from studies on the roles of two atypical response regulators (ARRs) in antibiotic biosynthesis. The CSR activator JadR1, an OmpR-type ARR of *S. venezuelae*, activates the expression of jadomycin B (JdB) biosynthetic genes in the presence of a low concentration of JdB and its homologs, but high JdB concentrations cause the dissociation of JadR1 from its target promoters. Thus, JdB interacts with JadR1 directly in a dose-dependent manner to control JdB production in a feedback regulatory mechanism (Wang et al., 2009). Similarly, a NarL-type ARR RedZ was shown to promote RED biosynthesis in *S. coelicolor* by activating the CSR activator gene *redD*, and the DNA-binding activity of RedZ to its target gene was released by adding RED (Wang et al., 2009). Other than the end-product, studies also suggest that biosynthetic intermediates of antibiotics can also serve as autoregulatory molecules to modulate their own biosynthesis. For example, the TetR family regulator JadR^∗^ can respond to early jadomycin intermediates [2,3-dehydro-UWM6 (DHU) and dehydrorabelomycin (DHR)] and release its repression on *jadY* transcription to ensure the timely supply of cofactors for JadG to convert DHR to the late intermediate jadomycin A (JdA) in jadomycin biosynthesis (Zhang et al., 2013).

Inspired by studies on the regulation of jadomycin biosynthesis in *S. venezuelae*, more antibiotics and their biosynthetic intermediates have been found to serve as autoregulatory molecules to modulate their own biosynthesis in many other streptomycetes. For illustration purposes, we herein only highlight several recent findings. For example, ChlF1, a TetR family regulator involved in chlorothricin biosynthesis of *Streptomyces antibioticus*, was found to repress the transcription of *chlF1*, *chlG* (encoding a major facilitator superfamily transporter), and *chlK* (encoding a type II thioesterase) but activates *chlJ* (encoding acyl-CoA carboxyl transferase) by binding to their promoter regions. However, ChlF1 was disassociated from its target promoters in the presence of chlorothricin and its biosynthetic intermediates (demethyl salicyloyl chlorothricin and deschloro-chlorothricin), which directly interacted with the regulator in a concentration-dependent manner (Li et al., 2016). In another study, CalR3, a TetR family regulator involved in calcimycin biosynthesis of *Streptomyces chartreusis* NRRL 3882, was found to repress the transcription of *calR3* and its adjacent *calT* (encoding a putative transmembrane efflux pump protein of the MMPL family) by binding to their promoter regions. Similarly, both calcimycin and its biosynthetic intermediate (cezomycin) can act as ligands to dissociate CalR3 from its target promoters in a concentration-dependent manner (Gou et al., 2017; Wu et al., 2018). Other than TetR family regulators, *Streptomyces* antibiotic regulatory proteins (SARPs) family regulator was also found to regulate antibiotic biosynthesis in response to antibiotics and their biosynthetic intermediates. For example, NosP was found to regulate nosiheptide production in response to both the end-product nosiheptide (NOS) and biosynthetic intermediate (NOS-AC) (Li et al., 2018). It should be noted that such findings are not limited to the genus *Streptomyces*. One example comes from the actinomycete *Amycolatopsis mediterranei*, in which rifamycin B (the end-product of rifamycin biosynthesis) serves as an extracellular signaling molecule to regulate rifamycin export in a feedback mechanism (Lei et al., 2018). Another study showed that 2,4-diacetylphloroglucinol and its biosynthetic intermediate could serve as autoregulatory ligands to regulate its own biosynthesis in *Pseudomonas fluorescens* (Yan et al., 2017). These studies suggested that antibiotics and their biosynthetic intermediates could function as autoregulators to regulate their own biosynthesis, mainly through the modulation of the binding activity of CSRs to their target genes within their cognate gene clusters.

### Antibiotics as Cross-Regulators of the Biosynthesis of Other Antibiotics

Earlier studies suggest that CSRs of one biosynthetic pathway can also control the biosynthesis of disparate antibiotic biosynthetic pathways. For example, RedZ, CSR of the *red* gene cluster, modulates the production of RED as well as that of ACT and CDA (Huang et al., 2005). In another study, the candicidin CSR FscRI was found to control the biosynthesis of candicidin as well as antimycin, the product of a disparate BGC in *Streptomyces albidoflavus* S4 (previously known as *Streptomyces albus* S4) (McLean et al., 2016; Li et al., 2019). Similar observations were also made with coordinated production of geldanamycin and elaiophylin by GdmRIII in *Streptomyces autolyticus* CGMCC0516 (Jiang et al., 2017), production of cephamycin C and clavulanic acid by CcaR in *Streptomyces clavuligerus* (Santamarta et al., 2002), and production of jadomycin and chloramphenicol by JadR1 in *S. venezuelae* (Wang et al., 2009). Recent years see growing evidence that antibiotics can also act as autoregulators regulating other biosynthetic pathways. The first examples to be described involve pseudo GBL receptors. In *S. coelicolor*, ScbR2, represses the same target, *kasO*, as the SCBs-interactive ScbR. Although *scbR2* is linked to the gene cluster for coelimycin biosynthesis, ScbR2 also responds to the endogenous antibiotics ACT and RED, and thereby regulates the production of these antibiotics (Xu et al., 2010). In *S. venezuelae*, JadR2, a close homolog of ScbR2, modulates JdB biosynthesis via direct repression of *jadR1*. This repression is sensitive to the jadomycin end products. Likewise, although *jadR2* is linked to the *jad* gene cluster for jadomycin biosynthesis, JadR2 could also bind to the endogenous antibiotic chloramphenicol, the product of the distant *cml* biosynthetic gene cluster (Xu et al., 2010). These studies suggest that CSRs of one biosynthetic pathway can also control disparate antibiotic biosynthetic pathways in response to different antibiotics. The pseudo GBL receptor AvaR2 of *S. avermitilis* was as an important repressor of avermectin and avenolide biosynthesis. It directly repressed transcription of *aveR*, *aco*, its own gene and the other two GBL receptor homologous genes (*avaR1* and *avaR3*) (Figure 3; Zhu et al., 2016). Interestingly, DNA-binding activity of AvaR2 can be modulated by endogenous avenolide in a concentration-dependent manner, indicating that avenolide serves as ligands for both the genuine GBL receptor AvaR1 and the pseudo GBL receptor AvaR2. Furthermore, DNA-binding activity of AvaR2 can also be modulated by exogenous antibiotics JdB and aminoglycoside antibiotics such as apramycin, hygromycin B, kanamycin and streptomycin (Zhu et al., 2016). It would be interesting to examine if JdB from *S. venezuelae* can serve as signals to modulate avermectin production in *S. avermitilis* under physiological conditions.

## Signaling Molecules in the Improvement of Antibiotic Titers and the Construction of Genetic Circuits

As mentioned above, signaling molecules serve as elicitors for antibiotic production in *Streptomyces*. It is reasonable that signaling molecules can be used to improve antibiotic production through exogenous addition. However, due to the fact that natural GBLs are produced in very small quantities, it is impractical to collect sufficient amount of GBLs for the improvement of antibiotic production. Synthetic GBLs and their analogs are then used for this purpose. Early studies showed that addition of chemically synthesized VB-C enhanced virginiamycin production in *Streptomyces virginiae* (Yang et al., 1995, 1996). Similarly, addition of chemically synthesized SCB1 promoted ACT production in *S. coelicolor*. Synthetic GBL analogs are also an alternative (Yang et al., 2009). In two recent studies, the addition of synthetic 1,4-butyrolactone enhanced validamycin A production in *Streptomyces hygroscopicus* 5008 and bitespiramycin production in *Streptomyces spiramyceticus* WS1-195, respectively (Tan et al., 2013; Gao et al., 2019). These studies showed that exogenous addition of signaling molecules can be used as an effective strategy to increase metabolic titers of antibiotics. However, this method has limited application due to the fact that there are only a few natural and synthetic analogs available. Therefore, it is necessary to expand the reservoir of both natural and synthetic signaling molecules. Furthermore, there is a growing interest in the construction of GBL-based genetic circuits in heterologous systems, which has been summarized by Takano and colleagues in 2015 (Biarnes-Carrera et al., 2015). Recently, orthogonal regulatory circuits based on the *S. coelicolor* GBL system have been constructed in *E. coli* (Biarnes-Carrera et al., 2018), reinforcing the promising applications of GBL systems from *Streptomyces* as a regulatory tool for synthetic biology in heterologous systems.

## Signaling Molecules in the Discovery of Novel Natural Products

High-throughput DNA sequencing technologies have resulted in an explosion of microbial genome sequences. Genome sequencing of multiple well-known actinomycetes has revealed that they harbor a great number of BGCs that are predicted to direct the biosynthesis of diverse natural products (Nett et al., 2009). It has become clear that these enormous reservoir of uncharacterized BGCs can serve as an important source of novel bioactive compounds. However, most of these BGCs are not expressed efficiently or not at all under routine laboratory culturing conditions (Mao et al., 2018). Though the reason for the silence of these gene clusters is complicated and remains obscure, a lack of specific signaling molecules may be one of the contributing factors (Liu et al., 2013). Interestingly, antibiotics can be used as chemical elicitors to trigger the expression of these cryptic BGCs and expand the chemical diversity of natural products.

### Antibiotics as Chemical Elicitors for the Discovery of Novel Natural Products

In recent years, growing evidence suggests that antibiotics, at sub-inhibitory concentrations, can potentiate antibiotic production in multiple *Streptomyces* species. For example, lincomycin at a sub-inhibitory concentration resulted in an elevated expression of the CSR activator gene *actII-ORF4*, and therefore increased ACT overproduction in *S. coelicolor* (Imai et al., 2015). A recent study suggests that lincomycin produced profound changes in gene expression profiles of *S. coelicolor* (Ishizuka et al., 2018). In another example, a sub-inhibitory concentration of JdB (the angucycline from *S. venezuelae*) induced early RED production and premature differentiation (formation of sporulating aerial mycelium) in *S. coelicolor*. Other angucyclines were also found to elicit similar phenotypes (Wang W. et al., 2014). Examples also include perturbation of antibiotics production by ribosome-targeting antibiotics (thiostrepton, spectinomycin, and chloramphenicol) in multiple *Streptomyces* species (Tanaka et al., 2017; Wang et al., 2017). Furthermore, antibiotics can also be used as chemical elicitors for the discovery of novel natural products. For example, *S. lividans* 1326 grown in the presence of lincomycin at a sub-inhibitory concentration produced abundant antibacterial compounds that were absent in cells grown in lincomycin-free medium. Some of these antibacterial compounds were revealed as novel congeners of CDA (Imai et al., 2015). In another case, polyether antibiotics, including promomycin and closely related salinomycin, monensin, and nigercin, at sub−inhibitory concentrations elicited antibiotic production in multiple *Streptomyces* strains (Amano et al., 2010, 2011). These studies suggest that antibiotics have the potential to serve as chemical elicitors for the discovery of novel bioactive natural products.

### High-Throughput Elicitor Screening for the Discovery of Novel Natural Products

**FIGURE 4:**
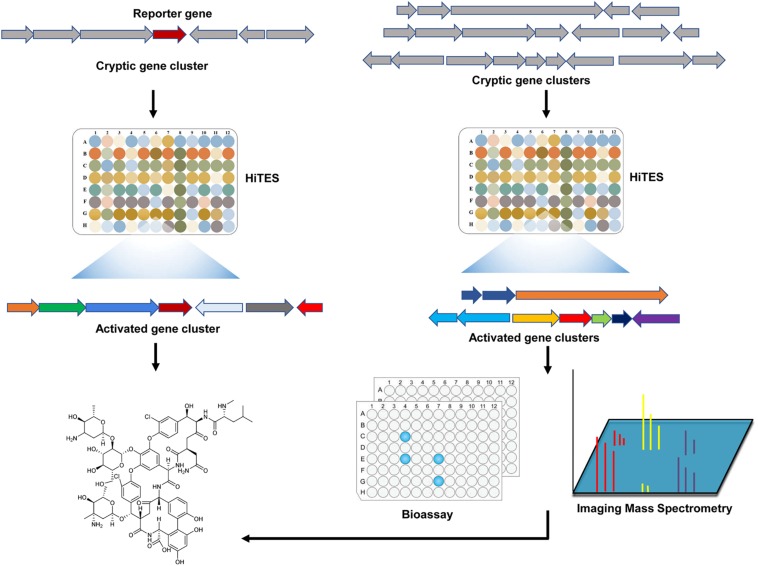
A general workflow of HiTES for the discovery of novel antibiotics. Initially, HiTES uses a reporter gene to allow a rapid read-out for the expression of the target cyptic gene cluster. The resulting reporter strain is then subjected to HiTES screening for the identification of novel antibiotics. Alternatively, microbial genomes encoding numerous cryptic gene clusters are directly subjected to HiTES screening. Cryptic gene clusters activated by small molecule elicitors are then subjected to high throughput bioassays for novel bioactive compounds. They can also be subjected to Imaging Mass Spectrometry to detect the natural products in a rapid and untargeted fashion. HiTES: high-throughput elicitor screening.

It is well-known that the cryptic gene clusters represent a treasure trove of new natural products, and various strategies and methods has been devised for the activation of these gene clusters (Liu et al., 2013; Niu et al., 2016; Niu, 2018; Niu and Li, 2019). Of special note is a chemogenetic method referred to as high-throughput elicitor screening approach (HiTES) (Figure 4). In this approach, a reporter gene is inserted into the BGC of interest to allow a rapid read-out for its expression. The resulting reporter strain is then screened against small molecule libraries to identify candidate elicitors (Seyedsayamdost, 2014). This approach was initially applied to activate two cryptic gene clusters in the Gram-negative bacterium *Burkholderia thailandensis*, leading to the identification of a new malleilactone analog. Interestingly, almost all elicitors identified from a library of 800 compounds were antibiotics (Seyedsayamdost, 2014). Furthermore, Seyedsayamdost and colleagues have employed this approach to identify 14 novel specialized products in *S. albus* J1074 (Xu et al., 2017). HiTES with *S. albus* J1074 using a library of ∼500 natural products identified two classical antibiotics (ivermectin and etoposide) as the best elicitors of a cryptic non-ribosomal peptide synthetase (NRPS) gene cluster (Xu et al., 2017). A major limitation of this method is that genetic manipulations and/or molecular biology approaches are required for the insertion of the reporter gene. To circumvent this problem, HiTES is then combined with bioactivity assays or imaging mass spectrometry (IMS) to identify novel antibiotics. In these approaches, a wild-type microorganism is subjected to HiTES. The resulting induced cultures are then screened directly for biological activity or subject to IMS (Figure 4). Application of these modified methods have uncovered two novel specialized products in *Streptomyces hiroshimensis* (Moon et al., 2019b), and many other novel specialized metabolites in diverse microorganisms (Moon et al., 2019a; Xu et al., 2019). These studies suggest that HiTES represents a promising new avenue for the discovery of novel bioactive natural products in a high-throughput manner.

## Conclusion and Perspectives

Over the past several decades, significant advances have been made in understanding the regulation of antibiotic biosynthesis in *Streptomyces*. The emerging picture shows a complex interplay of various signals and regulatory proteins. It is interesting to note that both hormone-like signaling molecules and antibiotics/their biosynthetic intermediates actively participate in the regulation of antibiotic production. These small molecule regulators exert their function through the modulation of the binding activity of different regulators to their target genes. Further studies are needed to reveal the complex interplay of autoregulators and multiple GBL receptor homologs in antibiotic biosynthesis of different *Streptomyces*. However, the number of hormone-like signaling molecules identified from *Streptomyces* is still limited, most likely due to the fact that they are produced in very small quantities. BLAST searches for homologs of AfsA from *S. griseus* within the actinobacteria genomes available from public databases indicated that AfsA-like proteins are present in most actinomycetes (Niu et al., 2016; Ahmed et al., 2017). Similar observations have been made with a BLAST search for homologs of Aco and Cyp17 proteins from *S. avermitilis* (Ahmed et al., 2017). It is strongly believed that GBLs and γ-butenolide autoregulators are widely distributed among actinomycetes. It is therefore necessary to identify more hormone-like signaling molecules. Currently, several approaches have been used to discover new signaling molecules in *Streptomyces*, including deletion of gene encoding repressor of signaling molecule biosynthetic gene (Sidda et al., 2016), bioassay using disruptants of known signaling molecule biosynthetic genes as indicator strains (Thao et al., 2017; Nguyen et al., 2018), and heterologous expression of putative signaling molecule biosynthetic genes in model microorganisms (Wang et al., 2018). These approaches will speed up the process of identifying new signaling molecules, and their signal transduction pathways. For autoregulators of antibiotics and their biosynthetic intermediates, more efforts should be geared toward understanding their effect on the fine-tuning regulation of antibiotic biosynthesis under physiological conditions. A better understanding of the complex regulatory pathways of signaling molecules in *Streptomyces* may be used to increase the metabolic titer of industrially and medically important antibiotics as well as activate silent antibiotic BGCs for the discovery of novel bioactive natural products in a wide range of actinomycetes.

## Author Contributions

All authors listed have made a substantial, direct and intellectual contribution to the work, and approved it for publication.

## Conflict of Interest

The authors declare that the research was conducted in the absence of any commercial or financial relationships that could be construed as a potential conflict of interest.
